# Association between serum folate concentrations and all-cause mortality in U.S. adults: a cohort study based on National Health and Nutrition Examination Survey III

**DOI:** 10.3389/fnut.2024.1408023

**Published:** 2024-07-10

**Authors:** Qingya Zhao, Xiaogang Lv, Qi Liu, Zhao Hu, Yiqiang Zhan

**Affiliations:** ^1^Department of Epidemiology, School of Public Health (Shenzhen), Sun Yat-Sen University, Shenzhen, China; ^2^Institute of Environmental Medicine, Karolinska Institutet, Stockholm, Sweden

**Keywords:** folic acid, mortality, NHANES, cohort, folate

## Abstract

The association between serum folate and all-cause mortality in general population remains unclear. The objective of this study was to investigate the potential association between serum folate concentrations and all-cause mortality in a large, prospective, long-term U.S. cohort. Our study included adults from the National Health and Nutrition Examination Survey (NHANES) III, and mortality data was obtained by linking with the National Death Index (NDI) until December 31, 2019. Cox proportional hazard models were used to calculate hazard ratios (HR) and 95% confidence intervals (CI) to assess the association between serum folate concentrations and all-cause mortality. A total of 12,862 participants were included in this cohort study. After a median follow-up of 26.4 years [interquartile range (IQR), 15.4–28.7 years], a total of 5,299 deaths were recorded. The risk of death was lower by 12% per 1.0 g/L increase in log-transformed serum folate concentrations (HR, 0.88; 95% CI, 0.83–0.94). Compared with the lowest quartiles of serum folate level, the risk of death was lower in the second (HR, 0.84; 95% CI, 0.72–0.97), third (HR, 0.78; 95% CI, 0.68–0.91) and the highest quartiles (HR, 0.78; 95% CI, 0.69–0.88) in multivariable-adjusted model. In subgroup analyses, the inverse association between serum folate and all-cause mortality remained statistically significant for women, men and non-Hispanic White people. Higher serum folate levels were found to be significantly associated with reduced risk of all-cause mortality. However, further studies are needed to verify these findings and explore the underlying mechanism.

## 1 Introduction

Folate, also referred to as vitamin B9, serves as a coenzyme or cosubstrate in single-carbon transfers involved in nucleic acids synthesis and amino acid metabolism (1). One of the crucial reactions dependent on folate is the conversion of homocysteine to methionine in the synthesis of S-adenosyl-methionine (SAM). This conversion plays a vital role in preventing the accumulation of homocysteine, which is a potential risk factor for various chronic diseases including cardiovascular diseases (CVD) (2, 3) and stroke (4, 5). Another reaction dependent on folate is the methylation of deoxyuridylate to thymidylate during Deoxyribonucleic acid (DNA) formation. This reaction is essential for accurate cell division and is believed to play a role in neural tube defects (6–8), megaloblastic anemia (9), and tumorigenesis (10, 11). Qualitative analysis revealed significant correlations between one-carbon metabolism nutrients and DNA methylation. Supplementation with folic acid alone or in combination with vitamin B12 significantly enhanced global DNA methylation in studies utilizing liquid chromatography-mass spectrometry, which exhibited markedly lower heterogeneity compared to other methods (12).

Research has evaluated the potential impact of folate on cancer risk with conflicting findings. Studies have demonstrated increased risk, no effect, and decreased risk (13–15). Many countries have implemented flour fortification programs by adding folic acid. Providing further insights into the full impact of folate on human health may provide more specific recommendations for policy makers. Moreover, many of these studies focused on specific populations or performed secondary analyses. For example, Li et al. identified a negative relationship between dietary folate intake and all-cause mortality in patients diagnosed with breast cancer (16). A study conducted on a Japanese cohort consisting of 3,050 adults found that higher serum folate concentrations were associated with lower risks of all-cause mortality. However, no significant associations were observed for CVD, respiratory, or cancer mortality (17). Conflicting with previous studies, a study indicated that dietary folate showed no significant association with either all-cause mortality or cancer-specific mortality among participants with cancer (18). A recent meta-analysis of randomized clinical trials (RCT) indicated that there were no significant relationships between folate supplementation and all-cause or CVD mortality. Furthermore, some RCTs even showed that folate supplementation was associated with elevated rates of all-cause mortality (19–22).

Thus, large-scale cohort studies are necessary to yield reliable results applicable to the general population. This study aims to establish the potential association between serum folate levels and all-cause mortality in the general U.S. population, using data obtained from a nationally representative sample of the National Health and Nutrition Examination Survey (NHANES).

## 2 Methods

### 2.1 Study design and population

Participants in this study were those who completed a study interview and examination as part of the NHANES III. The NHANES is a federally conducted survey in the United States that aims to provide representative data on the health and nutrition status of the noninstitutionalized population, which is conducted by the National Center for Health Statistics of the Centers for Disease Control and Prevention. Information on demographic and socioeconomic characteristics, health-related behaviors and health conditions of participants were collected with household interviews. Physical measurements and laboratory tests were administered by trained laboratory technicians in mobile examination centers (MEC). The details of the sampling methods and procedures have been published elsewhere. A total of 33,994 participants were randomly selected and included in NHANES III. Participants who had missing information on serum folate or unreliable serum folate concentration (serum folate concentration <0.1 ng/mL or >100.0 ng/mL, or red blood cell (RBC) folate concentration >6.0 ng/mL or <1000.0 ng/mL) were excluded from the analysis (*n* = 10,293). Those who were younger than 18 years old or older than 90 years old (*n* = 7,053) were also excluded from the study. Participants having extreme body mass index (<18 or >30) and concurrently presenting extreme triceps skinfold thickness (<9.9 or >19.8 for females, <7.5 or >15 for males) (*n* = 3,786) were excluded, resulting in a final sample size of 12,862 participants.

NHANES is a public database and all participants provided a written informed consent, consistent with approval from the National Center for Health Statistics Research Ethics Review Board (NCHS ERB).

### 2.2 Serum folate measurement

Blood specimens of participants were collected in the MEC by trained laboratory technicians, and frozen specimens were shipped overnight according to the protocol. Serum folate concentrations were determined by the National Center for Environmental Health at the Centers for Disease Control and Prevention using a commercially available radioprotein binding assay kit (Quantaphase II, Bio-Rad Laboratories).

### 2.3 Outcome measurement

Mortality status was ascertained by linking the NDI public-access files up until December 31, 2019. All-cause mortality was defined as death due to any cause. Follow-up duration was calculated from the date of examination to the date of death or the end of 2019, whichever occurred first.

### 2.4 Potential confounders

Demographic information, including age at baseline, sex (men or women), education, ethnicity (non-Hispanic White people, non-Hispanic Black people, Hispanic American, or others), and military background (ever served in armed forces, never served in armed forces) were collected through a standard questionnaire by trained interviewers using a computer-assisted personal interviewing system. Educational attainment was classified as below high school (<9), high school (9–12), college or more (>15) in this study. Participants who had a physician-diagnosed history of diabetes or were taking diabetes pills or had a glycated hemoglobin value ≥6.5% or a fasting plasma glucose ≥7.0 are classified as diabetic. Those with an average systolic blood pressure ≥140 mmHg or diastolic blood pressure ≥90 mmHg were classified as hypertensive.

### 2.5 Statistical analysis

Descriptive characteristics at baseline are presented as mean with standard deviation (SD) for quantitative variables and as frequencies and percentages for categorical variables. Serum folate concentrations were categorized into quartiles after natural logarithmic transformation, and the first quartile was selected as the reference group. The survival rate function was estimated, and survival curves according to quartiles of serum folate showed significant differences by the log rank test. Adjusted for weights that account for the complex survey design (including oversampling, survey nonresponse, and post-stratification), cox proportional hazard regression models were employed to calculate the hazard ratios and corresponding 95% confidence intervals. Schoenfeld residuals were used to test the proportional hazards assumption, and no violation was observed. We constructed three cox models for the analysis. Beyond the unadjusted model, our multivariable models incorporated adjustments for age, sex, ethnicity, educational level, diabetes, and hypertension. Folate concentrations were examined as a continuous variable in Model 2 to assess the impact per one-unit change, and as quartiles in Model 3. Furthermore, we conducted stratified analyses by sex and ethnicity to assess potential effect modifications.

All analyses were conducted following the NHANES III analytical guidelines and were performed with R 4.2.3. Statistical significance was tested at *p* < 0.05, and all tests were 2-tailed.

## 3 Results

### 3.1 General characteristics

Of 12,862 participants included in this study, the mean (SD) age was 47.5 (20.4) years old and 6,232 (48.5%) were women. The general characteristics of the participants by sex are presented in Table 1. Compared with men participants, women participants were more likely to be younger, non-Hispanic White people, more educated, never served in armed forces and tended to have lower prevalence rates of hypertension.

**Table 1:** Baseline characteristics of the study population.

Variable	Men (*n* = 6,630)	Women (*n* = 6,232)
Age (year)	47.9 ± 20.2	47.1 ± 20.6
Race/Ethnicity, *n* (%)		
Non-Hispanic White people	2,730 (41.2%)	2,830 (45.4%)
Non-Hispanic Black people	1,704 (25.7%)	1,542 (24.7%)
Mexican-American	1,944 (29.3%)	1,583 (25.4%)
Other Hispanic	252 (3.8%)	277(4.4%)
Education, *n* (%)		
<High school	1,836 (27.7%)	1,440 (23.1%)
High school	2,807 (42.7%)	2,832 (45.4%)
Some college or more	1,919 (28.9%)	1,914 (30.7%)
Uncertain*	68 (1.0%)	46 (0.7%)
Military, *n* (%)		
Ever served in armed forces	1,948 (29.4%)	1,163 (18.7%)
Never served in armed forces	4,632 (69.9%)	5,036 (80.8%)
Uncertain*	50 (0.8%)	33 (0.5%)
Diabetes, *n* (%)	734 (11.1%)	629 (10.1%)
Hypertension, *n* (%)	1,572 (23.7%)	1,305 (20.9%)

^*^Categorized based on self-report in the NHANES interview.

### 3.2 Associations between serum folate with all-cause mortality

**Figure 1:**
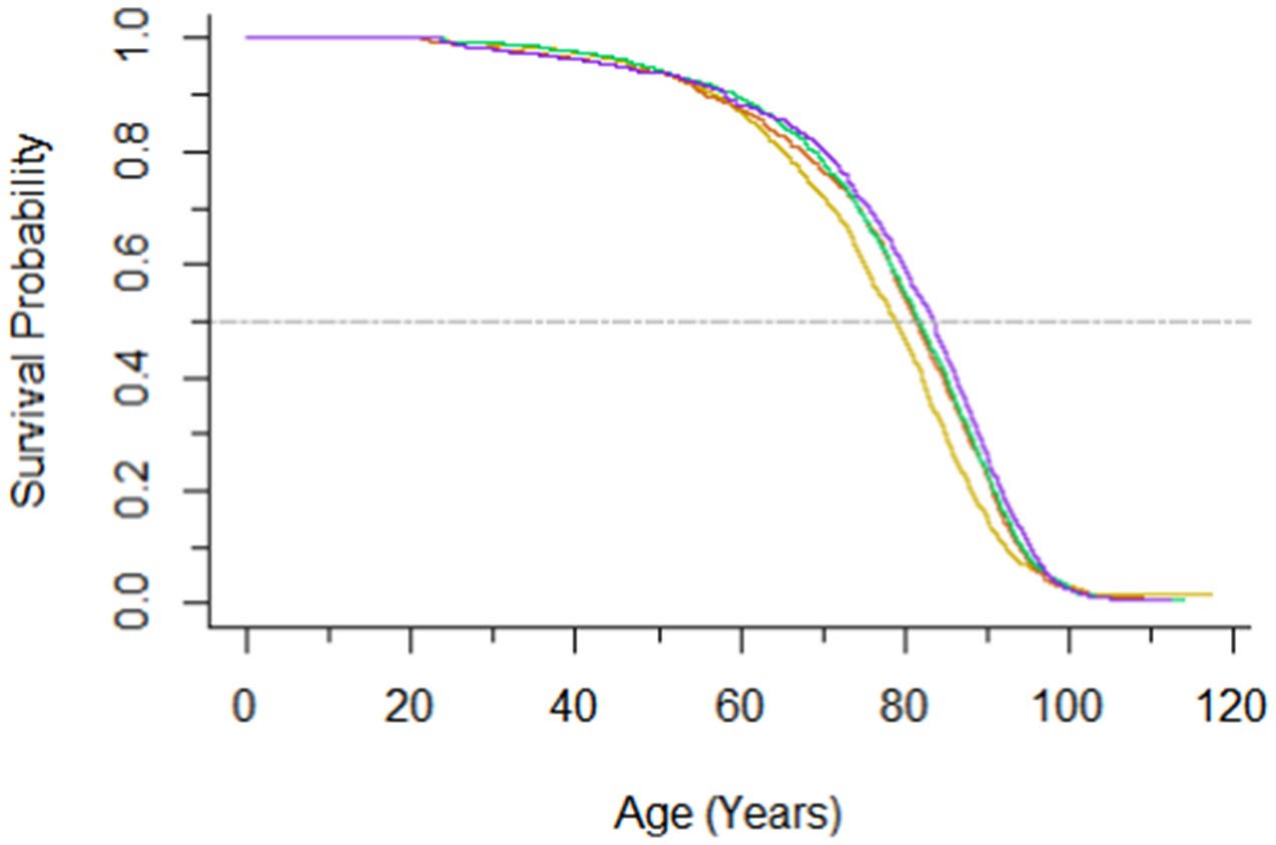
Survival curve according to the quartiles of serum folate.

During a median follow-up of 26.4 (IQR, 15.4–28.7) years, a total of 5,299 deaths were recorded. Hazard ratios for all-cause mortality according to serum folate were presented in Table 2. An inverse association between serum folate and all-cause mortality was observed. Participants with per 1.0 μg/L increase in log-transformed serum folate concentrations were associated with a 12% decreased risk of all-cause mortality after adjusting for age, sex, ethnicity, educational level, diabetes and hypertension (HR, 0.88; 95% CI, 0.83–0.94). The crude HRs of all-cause mortality were 0.83 (95% CI, 0.70–0.99), 0.75 (95% CI, 0.63–0.88), and 0.72 (95% CI, 0.63–0.83) for the second, third and highest quartiles of folate, respectively. After multivariable adjustment, compared with individuals in the first quartile of serum folate, those in the second, the third, and the highest quartile had 0.84 (95% CI, 0.72–0.97), 0.78 (95% CI, 0.68–0.91), and 0.78 (95% CI, 0.69–0.88) -times lower risk of all-cause mortality. The survival curves are presented in Figure 1.

**Table 2:** Hazard ratio (HRs) and 95% CIs for all-cause mortality according to serum concentrations of folate.

Folate/(μg L^−1^)*	HR (95% CI)	HR (95% CI)a	HR (95% CI)a
Q1 (−0.92, 1.28]	1.00	–	1.00
Q2 (1.28, 1.70]	0.83 (0.70–0.99)	–	0.84 (0.72–0.97)
Q3 (1.70, 2.15]	0.75 (0.63–0.88)	–	0.78 (0.68–0.91)
Q4 (2.15, 4.55]	0.72 (0.63–0.83)	–	0.78 (0.69–0.88)
Continuous	–	0.88 (0.83–0.94)	–

^*^Natural logarithmic transformed.

a Adjusted for age, sex (male or female), ethnicity (non-Hispanic White people, non-Hispanic Black people, Mexican-American, or other), educational level (below high school, high school, college or more), diabetes (yes or no) and hypertension (yes or no).

### 3.3 Stratified analyses

The results of the subgroup analyses are presented in Table 3. The association between serum concentrations of folate and all-cause mortality was attenuated when stratified by sex. The crude HRs and adjusted HRs remained significant for the third and the highest quartiles, whereas that for the second quartile failed to reach statistical significance in both sexes.

**Table 3:** Hazard ratio (HRs) and 95% CIs for all-cause mortality according to serum concentrations of folate, stratified by gender and ethnicity.

	Folate/(μg L^−1^)*	HR (95% CI)	HR (95% CI)a	HR (95% CI)b
Gender				
Male (*n* = 6,630)	Q1 (−0.92, 1.28]	1.00	–	1.00
	Q2 (1.28, 1.70]	0.86 (0.72, 1.03)	–	0.85 (0.72, 1.00)
	Q3 (1.70, 2.15]	0.76 (0.62, 0.94)	–	0.79 (0.65, 0.95)
	Q4 (2.15, 4.55]	0.79 (0.67, 0.94)	–	0.82 (0.70, 0.94)
	Continuous	–	0.89 (0.83, 0.96)	–
Female (*n* = 6,232)	Q1 (−0.92, 1.28]	1.00	–	1.00
	Q2 (1.28, 1.70]	0.80 (0.63, 1.01)	–	0.80 (0.64, 1.01)
	Q3 (1.70, 2.15]	0.76 (0.64, 0.90)	–	0.78 (0.65, 0.93)
	Q4 (2.15, 4.55]	0.72 (0.61, 0.84)	–	0.74 (0.63, 0.87)
	Continuous	–	0.88 (0.81, 0.96)	–
Ethnicity				
Non-Hispanic White people (*n* = 5,560)	Q1 (−0.92, 1.28]	1.00	–	1.00
	Q2 (1.28, 1.70]	0.80 (0.70, 0.91)	–	0.80 (0.71, 0.91)
	Q3 (1.70, 2.15]	0.73 (0.62, 0.85)	–	0.76 (0.66, 0.88)
	Q4 (2.15, 4.55]	0.70 (0.60, 0.80)	–	0.75 (0.66, 0.85)
	Continuous	–	0.87 (0.82, 0.93)	–
Non-Hispanic Black people (*n* = 3,246)	Q1 (−0.92, 1.28]	1.00	–	1.00
	Q2 (1.28, 1.70]	0.99 (0.84, 1.17)	–	0.97 (0.83, 1.15)
	Q3 (1.70, 2.15]	0.93 (0.79, 1.09)	–	0.90 (0.76, 1.06)
	Q4 (2.15, 4.55]	0.92 (0.75, 1.12)	–	0.88 (0.73, 1.07)
	Continuous	–	0.92 (0.81, 1.04)	–
Mexican-American (*n* = 3,527)	Q1 (−0.92, 1.28]	1.00	–	1.00
	Q2 (1.28, 1.70]	0.81 (0.61, 1.07)	–	0.75 (0.56, 1.01)
	Q3 (1.70, 2.15]	0.88 (0.73, 1.06)	–	0.87 (0.69, 1.09)
	Q4 (2.15, 4.55]	0.66 (0.56, 0.77)	–	0.70 (0.58, 0.84)
	Continuous	–	0.84 (0.74, 0.95)	–
Other (*n* = 529)	Q1 (−0.92, 1.28]	1.00	–	1.00
	Q2 (1.28, 1.70]	0.99 (0.54, 1.80)	–	1.04 (0.60, 1.81)
	Q3 (1.70, 2.15]	0.72 (0.27, 1.95)	–	0.73 (0.30, 1.77)
	Q4 (2.15, 4.55]	0.90 (0.47, 1.73)	–	0.78 (0.42, 1.45)
	Continuous	–	0.90 (0.64, 1.26)	–

*Natural logarithmic transformed.

a Adjusted for age, sex (male or female), ethnicity (non-Hispanic White people, non-Hispanic Black people, Mexican-American, or other), educational level (below high school, high school, college or more), diabetes (yes or no) and hypertension (yes or no). The strata variable was not included when stratifying by itself.

b Adjusted for age, sex (male or female), ethnicity (non-Hispanic White people, non-Hispanic Black people, Mexican-American, or other), educational level (below high school, high school, college or more), diabetes (yes or no) and hypertension (yes or no). The strata variable was not included when stratifying by itself.

However, we can observe a statistically significant association between serum folate and all-cause mortality only for non-Hispanic White people. The crude HRs and adjusted HRs for increasing quartiles and continuous concentrations of serum folate remained statistically significant for non-Hispanic White people. In addition, for Mexican-Americans, the HRs for continuous concentrations of folate and for the fourth quartile were also significant. As for non-Hispanic Black people and other ethnicities, no statistical significance was observed in the three models.

## 4 Discussion

In this large, prospective cohort study of adults in the U.S., we observed a significant association between serum folate and all-cause mortality. Briefly, higher serum folate was significantly associated with lower all-cause mortality risk in general population. This inverse association remained statistically significant for women, men and non-Hispanic White people. However, in subgroup analyses this association became weaker among Mexican-Americans and nonsignificant for non-Hispanic Black people, and individuals of other ethnicities.

As is reported, ethnicity is one of the significant determinants of RBC and serum folate concentrations. Compared with the White people, RBC and serum folate concentrations were significantly lower in the Black people (23). Another study, based on the NHANES 1999–2016, found similar results. The non-Hispanic White people showed higher folate concentrations compared to the non-Hispanic Black people and the Hispanic (24). In addition to folic acid intake from fortification and supplements, blood folate concentrations depend on folate intake from natural foods and genetic variation in the population. Variations in dietary habits, demographic characteristics, and specific genes like the MTHFR variant are significant contributors to the effects of folate across different ethnicities. In addition, the “other” ethnicities may not have had sufficient power due to smaller sample sizes in this study.

### 4.1 Comparison with previous studies

Our findings are consistent with several previous studies that have demonstrated an inverse association between folate levels and all-cause mortality. In a prospective cohort conducted in Israel, it was observed that older adults with serum folate deficiency (<4.4 ng/mL) had an increased risk of dementia and nearly triple the risk of all-cause mortality (25). Another study conducted in Western Australia also found negative associations between serum folate and RBC folate levels with certain types of cancer risk, indicating that protective effects of folate on cause-specific mortality (26). Bo et al. found that intake of dietary folate, which was assessed by 24 h diet recall, was in relation to all-cause mortality and CVD mortality (27). This study measured folate exposure by another method, but came to the same conclusion, providing more convincing evidence for the inverse association between folate and mortality.

However, some other studies reported inconsistent results. A 10 year cohort study found that there was no significant association between either serum folate (Adjusted HR, 1.03; 95% CI, 0.91–1.16) or RBC folate (Adjusted HR, 1.16; 95% CI, 0.99–1.36) with mortality risk among hypertensive patients with elevated homocysteine (28). Different from our study, it was focus on hypertensive patients with elevated homocysteine, resulting in a small sample of only 1,753 participants. This small sample size may not have enough power to find such an association. Indeed, two randomized, double-blind, placebo-controlled clinical trials, including 6,837 participants, reported that treatment with folate plus vitamin B12 was even associated with increased cancer outcomes and all-cause mortality in patients with ischemic heart disease in Norway (19). In general, the conflicting results observed in these studies can be attributed to several factors, including variations in the measurement of exposure, differences in study populations and regions. Furthermore, inconsistencies can also be influenced by sample size and duration of follow-up.

### 4.2 Potential mechanisms

Although the mechanisms underlying how a low level of folate status contributes to mortality remain unclear, several possible explanations can be considered. A hypothesis pertains to alterations in DNA methylation. Folate plays a crucial role in the synthesis of SAM, the principal methyl donor in most cellular reactions, including the methylation of cytosine in the DNA molecule (29). Regional hypomethylation is associated with alterations in chromatin conformation and with alterations in interaction between DNA and methyl-specific proteins, promoting genomic instability (30). Folate deficiency disturbs cytosine methylation, leading to global DNA hypomethylation and/or changes in gene-specific methylation and inappropriate protooncogene activation (30, 31). Within the methionine cycle, 5-methyltetrahydrofolate methylates homocysteine to methionine, preventing the continued buildup of homocysteine (32). Folate deficiency leads to an increase in blood homocysteine concentrations, and an elevated level of homocysteine concentrations has shown a significant association with the incidence of various diseases, such as heart disease (33, 34), tumorigenesis (35, 36), stroke (37, 38), and diabetes mellitus (39). Another hypothesis pertains to how folate deficiency disrupts the synthesis and repair of DNA. Folate is critical for the synthesis of both purines and the pyrimidine nucleoside thymidine. Deoxyuridine monophosphate is converted to thymidine monophosphate with 5,10-methylenetetrahydrofolate as the methyl donor (40). In addition, 5,10-formyltetrahydrofolate is involved in the production of both adenosine and guanosine (41, 42). The production of these DNA precursors is essential for normal DNA synthesis and repair. When folate levels are limited, it leads to an imbalance in purine and pyrimidine DNA precursors, ultimately inhibiting normal DNA repair processes. Additionally, uracil, an atypical component in DNA, is mistakenly inserted into the DNA molecule instead of thymidine, leading to DNA strand breakage, chromosomal damage, and malignant transformation (43). Thus, low levels of folate status may be associated with tumorigenesis (41), aging (2), and related diseases (7, 8). Due to the above mechanisms, low level of folate may be associated with various related diseases and mortality, and mechanistic studies are warranted to clarify the roles of serum folate in the long-term health of individuals.

### 4.3 Strengths and limitations

The strengths of our study are its prospective design, long-term follow-up duration, and large national representative samples, adjusted for weights to account for the complex survey design, survey nonresponse, and post-stratification. In addition, the measurements of serum folate concentrations and covariates are generally reliable because they were under the well-designed guidance of NCHS of the United States, a professional national organization.

There are some limitations to our study as well. First, the measurement of circulating folate was merely based on a single serum examination at baseline, which may not reflect the long-term folate status accurately. Second, the mortality outcomes of participants were determined by linkage to the NDI through a probabilistic match, which might result in misclassification. Third, residual or unmeasured confounding cannot be entirely excluded. In addition, we failed to consider whether participants have used folic acid supplements due to insufficient data. Although few prospective studies have examined the association between serum folate levels and all-cause mortality in general population, the observational design of our study does not allow us to make causal inference from the results.

It is important to address the limitation related to the measurement of dietary adherence using the Perceived Dietary Adherence Questionnaire. This limitation stems from the unavailability of data on the questionnaire in the NHANES III public database, which restricted the direct assessment of dietary adherence through this specific tool. Therefore, future studies aiming to explore the relationship between dietary adherence and health outcomes should consider incorporating more detailed dietary assessment methods to provide a comprehensive understanding of dietary patterns and their impact on various health parameters.

The inverse association observed between folate intake and mortality suggests that maintaining a healthy diet is crucial for overall health. Consuming foods rich in folate, such as vegetables, animal liver, and egg yolks, may offer protection against related diseases. Nevertheless, it is worth noting that our study focused solely on the nutrient folate. Some studies have highlighted the role of dietary patterns rather than single nutrient as crucial factors in chronic diseases (44, 45). Lifestyle risk factors such as unhealthy diet, lack of physical activity, smoking, and excessive alcohol consumption are correlated and tend to co-occur with other behaviors within the population (46). Additionally, due to the intricate and interactions between nutrients, focusing on a single nutrient may underestimate its impact on health. Recently, researchers have attempted to cluster lifestyle risk factors together. The combination of two or more unhealthy lifestyle behaviors is generally linked to a higher risk of disease than would be expected from each individual risk factor alone (47, 48). Therefore, our study may underestimate the effect of folate, and more detailed comprehensive assessments of multiple nutrients, dietary habits, and other lifestyle habits are needed.

## 5 Conclusion

In this large, prospective cohort study, we found a significant inverse association between serum folate and overall mortality risk. Specifically, higher serum folate was associated with a reduced risk of all-cause mortality. Nevertheless, further studies are necessary to fully comprehend the underlying mechanisms of folate on mortality risk.

## Data availability statement

Publicly available datasets were analyzed in this study. This data can be found here: NHAMES (https://www.cdc.gov/nchs/nhanes/index.htm).

## Ethics statement

The studies involving humans were approved by School of Public Health, Sun-Yat-Sen University. The studies were conducted in accordance with the local legislation and institutional requirements. The participants provided their written informed consent to participate in this study.

## Author contributions

QZ: Conceptualization, Formal analysis, Writing – original draft, Writing – review & editing. XL: Validation, Writing – review & editing, Methodology. QL: Validation, Writing – review & editing, Methodology. ZH: Validation, Conceptualization, Supervision, Writing – review & editing, Writing – original draft. YZ: Validation, Conceptualization, Supervision, Writing – review & editing, Writing – original draft.
